# Design Principles for Nanoparticle Plasmon-Enhanced Organic Solar Cells

**DOI:** 10.1186/s11671-018-2620-4

**Published:** 2018-07-16

**Authors:** Juanjuan Wang, Shengli Jia, Yang Cao, Wenhao Wang, Peng Yu

**Affiliations:** 1 Department of Chemistry and Chemical Engineering, Luliang University, Lvliang Shanxi, 033001 People’s Republic of China; 2 State Key Laboratory of Electronic Thin Film and Integrated Devices, University of Electronic Science and Technology of China, Chengdu, 610054 People’s Republic of China; 3 Institute of Fundamental and Frontier Science, University of Electronic Science and Technology of China, Chengdu, 610054 People’s Republic of China

**Keywords:** Organic solar cell, Plasmonic, Scattering, Field enhancement, FDTD, Nanoparticle

## Abstract

Plasmonic metallic nanoparticles are coupled to the organic solar cells to overcome the trade-off between the light absorption and carrier collection. They are usually located inside or outside of the active layers. However, no detailed comparison was reported on the light absorption difference when nanoparticles are located inside or outside of the active layers. In this paper, we compare light-trapping abilities of Ag nanospheres in organic solar cells when they are located inside and outside of the photoactive layer. We show that large-sized nanoparticles are preferred when they are placed outside of the active layer while small-sized nanoparticles are favored in the case of embedding nanoparticles in the homogenous active layer.

## Background

Organic solar cells (OSCs) are ideal candidates to replace mainstream inorganic solar cells for achieving cost-effective photovoltaics (PVs) due to advantages of OSCs including lightweight, low-cost, low-temperature fabrication process, semi-transparency, and mechanical flexibility [1, 2]. Recent progress of OSCs has demonstrated over 10% power conversion efficiency (PCE) based on single-junction devices, which put them in direct competition with their Si and GaAs counterparts. Single-junction terrestrial OSCs achieved an efficiency of 11.2 ± 0.3 measured under the global AM1.5 spectrum (1000 W/m^2^) at 25 °C [2]. A polymer enables a solution-processed tandem solar cell with certified 10.6% power conversion efficiency under standard test conditions [3]. A new polymer donor (PBDB-T-SF) and a new small molecule acceptor (IT-4F) for fullerene-free OSCs were designed and synthesized, yielding a PCE of 13.1% [4]. Ternary organic solar cells offering a PCE of 14% was reported [5]. Although the efficiency has broken up to 10%, the whole scale market is not mature to compete with the Si solar cells. The key challenge in OSCs lies in boosting the efficiency above 10% under an industrial fabrication process. Due to the intrinsic low charge-carrier mobility and exciton diffusion properties of organic molecules, the thickness of OSCs is limited and thus curbs the light absorption of OSCs. To circumvent the trade-off between light absorption and carrier collection, during past decades, many light-trapping schemes have been proposed, such as quantum dot solar cell [6–9], nanowire solar cell [10, 11], and plasmonic solar cell [6, 12, 13]. The plasmonic solar cells provide a practical way to boost the light harvesting of solar cells while maintaining their carrier collection efficiency [14]. The noble metallic nanoparticles (NPs) incorporated in solar cells can improve efficiency by creating highly concentrated near-field, increasing pathlength via far-field scattering and waveguide coupling [15]. For example, previous simulations on plasmon-enhanced inorganic solar cells have adopted 10–100-nm-thick active layers for proof-of-concept demonstrations [12, 16–18].

In the mainstream OSCs design process, the plasmonic metallic NPs are located outside/inside the active layers. Embedding metallic NPs in active layers of OSCs exploits the strongly confined field of the localized surface plasmon resonance (LSPR) and more efficient light scattering within the active layers while introducing metallic NPs outside the active layer(s) can be accomplished by coupling the NPs on indium tin oxide (ITO) or inside a poly(3,4-ethylenedioxythiophene): poly (styrene sulfonate) (PEDOT: PSS) buffer layer. However, there is no comparison to distinguish the influence of plasmonic NPs when they are introduced into these two structures. In this paper, we compare light-trapping abilities of the NPs when they are placed inside and outside the active layers. Our work provides a design principle for NP plasmon-enhanced OSCs.

## Methods

**Fig. 1:**
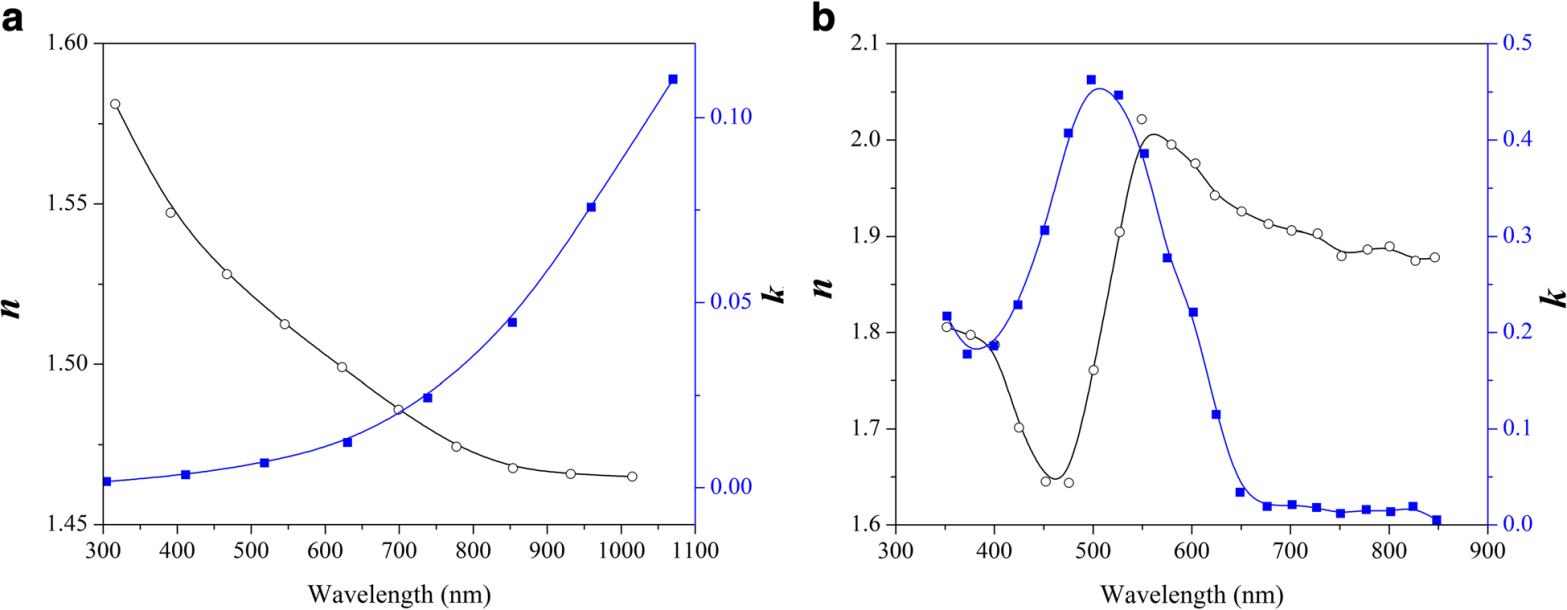
The complex refractive index ***n*** and extinction coefficient ***k*** of PEDOT: PSS **a** and P3HT: PCBM **b** in the FDTD simulation

All the simulations were carried out by using finite-difference time-domain (FDTD) method solving Maxwell’s equations. During the scattering cross-sectional simulations, a total-field scattered-field (TFSF) source in the wavelength ranging from 300 to 700 nm was injected into a box containing the NPs. Here, we choose the Ag in the simulation because its plasmonic resonance is well fitted to the absorption spectra of the P3HT:PCBM [19, 20]. Cathode Al material was taken from ref. [21]. The complex refractive index (***n***, ***k***) of ITO and Ag were fitted from refs. [21, 22], respectively. The ***n*** and ***k*** of PEDOT: PSS and a blend of poly(3-hexylthiophene) (P3HT) and [6,6]-phenyl-C61-butyric acid methyl ester (PCBM) were fitted from refs. [23, 24], respectively, as replotted in Fig. 1a, b. The thicknesses of ITO, PEDOT: PSS and P3HT: PCBM are 100, 40, and 200 nm, respectively. In our simulation, we choose the PEDOT: PSS as the buffer layer and the P3HT: PCBM as the active layer [14, 24]. The normalized scattering/absorption cross sections, *Q*_scat_/*Q*_abs_, are defined by scattering/absorption cross section divided by geometric cross section of NPs. The fraction of light scattered into the substrate, *f*_sub_, is defined as the power scattered towards the substrate divided by total scattered power. For Ag NPs, override mesh settings dx = dy = dz = 0.1, 0.5, and 1 nm were chosen for small- (5, 10 nm), medium- (20, 40 nm), and large-sized (60, 80 nm) NPs, respectively, while an automatic mesh generation method was used in other simulation regions. We define small- (5, 10 nm), medium- (20, 40 nm), and large-sized (60, 80 nm) NPs based on published experimental work [25, 26]. We decreased the mesh size and set perfectly matched layer (PML) parameters until the results were convergent.

## Result and Discussion

**Fig. 2:**
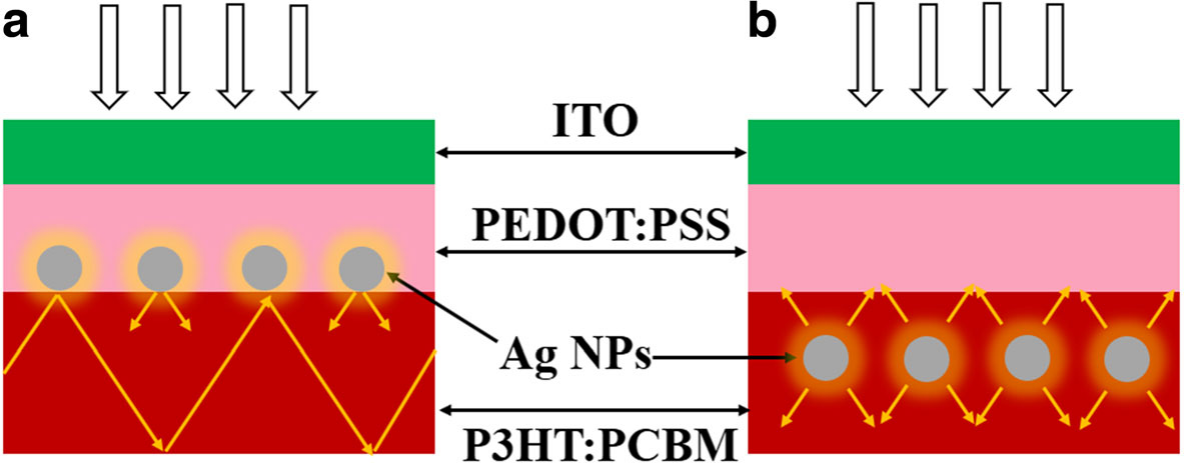
Schematic illustration of light trapping through scattering and local field enhancement in **a** NPs located outside of the active layer and **b** NPs located inside of the active layer

As shown in Fig. 2a, b, the architectures correspond to the case when Ag NPs are located inside or outside of the active layer. In the practical fabrication process, thickness of the PEDOT: PSS layer is ~ 50 nm and the active layer is ~ 200 nm. In our simulation, these two layers are set to be 40 and 200 nm, respectively. For NPs located within the active layer, it does not affect the calculated result when NPs are in homogenous material. For NPs located outside of the active layer, although many publications use NPs embedded in PEDOT:PSS totally, there are some publications use large NPs beyond thickness of the PEDOT: PSS layer [27, 28]. Therefore, it is applicable in experimental fabrication. The ***n*** of ITO and PEDOT: PSS (400–800 nm) is ~ 2.1–1.6 and ~ 1.55–1.45, respectively. The difference of ***n*** is not obvious. According to an approximation theory for absorption and scattering cross-sectional calculation [15], the differences in the cross sections between fully covered and partially covered NPs in PEDOT: PSS are fairly small. Therefore, we can conclude that it slightly affects the calculated results when NPs are larger than the thicknesses of PEDOT:PSS and active layers. The role of plasmonic effects of NPs for light trapping is also illustrated in the structures. Requirements of light-trapping ability are different between the two structures. In Fig. 2a, since the NPs are located outside of the active organic layer, the near-field enhancement has a limited impact on the absorption enhancement because only near-field at the bottom of NPs contribute to the absorption enhancement. Moreover, as the diameter increases, the near-field of large NPs penetrates a longer distance, away from the surface of the NPs [29]. As the diameter increases, the scattering effect will become stronger according to Mie theory. Therefore, it is unnecessary to take the near-field enhancement into account when NPs are located outside of the active layer. However, the NPs in the active layers benefit from near-field enhancement, increasing its effective absorption cross section and thus exciton dissociation, as shown in Fig. 2b.

### NPs Located Outside of the Active Layers

**Fig. 3:**
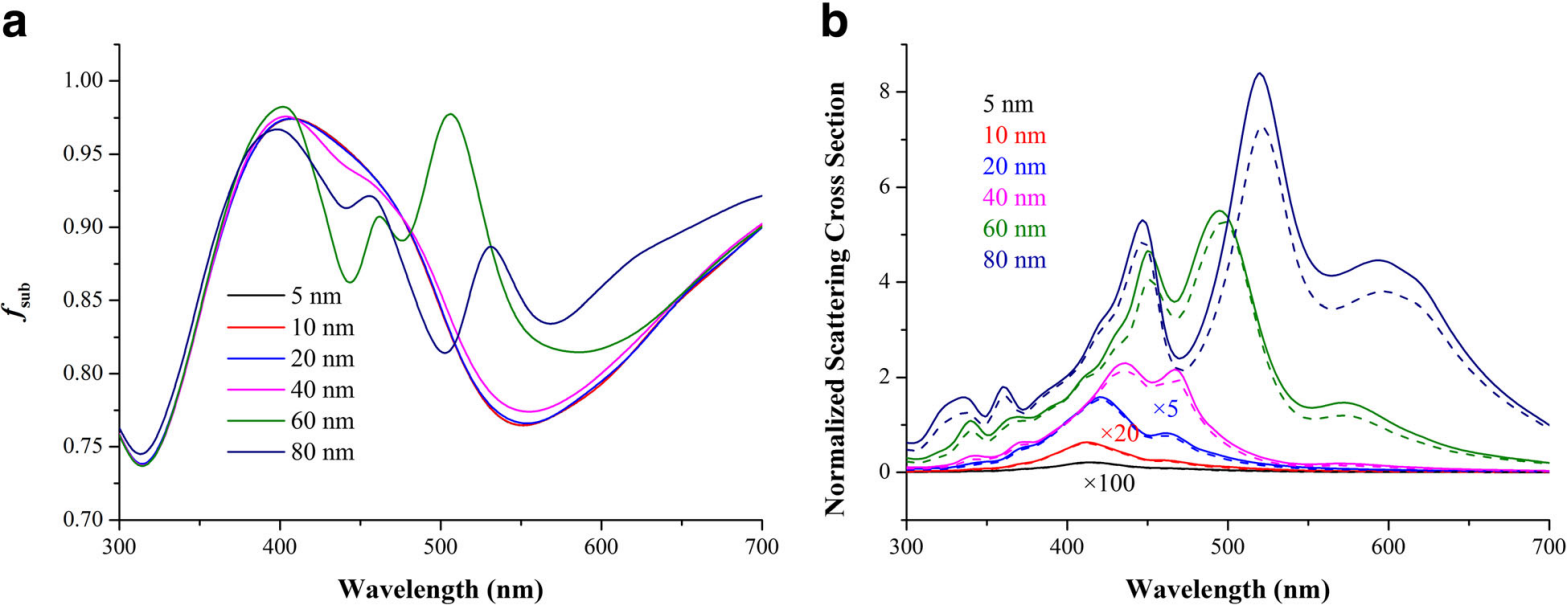
**a** The fraction of light scattered into the active layer. **b** Total scattering cross section (solid), cross section for light scattered into the substrate (dashed line), all normalized to geometrical cross section

Although light absorption enhancement in plasmonic NP-based OSCs is observed, the bare metallic NPs in the active layer also induce charge recombination and exciton quenching near the vicinity of metal surface due to dipole-dipole and charge-trapping coupling [12, 30]. As size increases, charge recombination and exciton quenching will become more serious [31, 32]. In order to suppress the exciton quenching and charge-trapping effect of NPs, three remedies can be introduced: coating a thin dielectric layer on metallic NPs [12, 30], forming NPs by laser ablation in liquids [33], and placing NPs outside active layer [14, 28]. As discussed in the above section, when NPs are located outside of the active layers, the scattering properties of NPs are critical for light trapping. Therefore, the fraction of light scattered underneath the active layer, *f*_sub_, is compared among the NPs with different diameters as shown in Fig. 3a. The trends of *f*_sub_ increase as the size of NPs increases, which disagrees with the Ag NPs located on the Si surface as calculated in ref. [17]. As can be seen from Fig. 3a, an evident dip occurs at ~ 550 nm, meaning a large amount of light is scattered towards the back and is wasted. However, it is difficult to judge scattering contributions of different-sized NPs because they have different *f*_sub_ values. Therefore, we plot the total *Q*_scat_ and *Q*_scat_ for light scattered into the substrate, as shown in Fig. 3b. As size increases, the total *Q*_scat_ of large-sized NPs have large values which exceed that of the medium- and small-sized NPs within a broadband spectrum. The large-sized NPs with large scattering *Q*_scat_ behave as effective subwavelength scattering elements that couple and trap sunlight into the photoactive layer and thus the optical pathlength is enhanced [16].

**Fig. 4:**
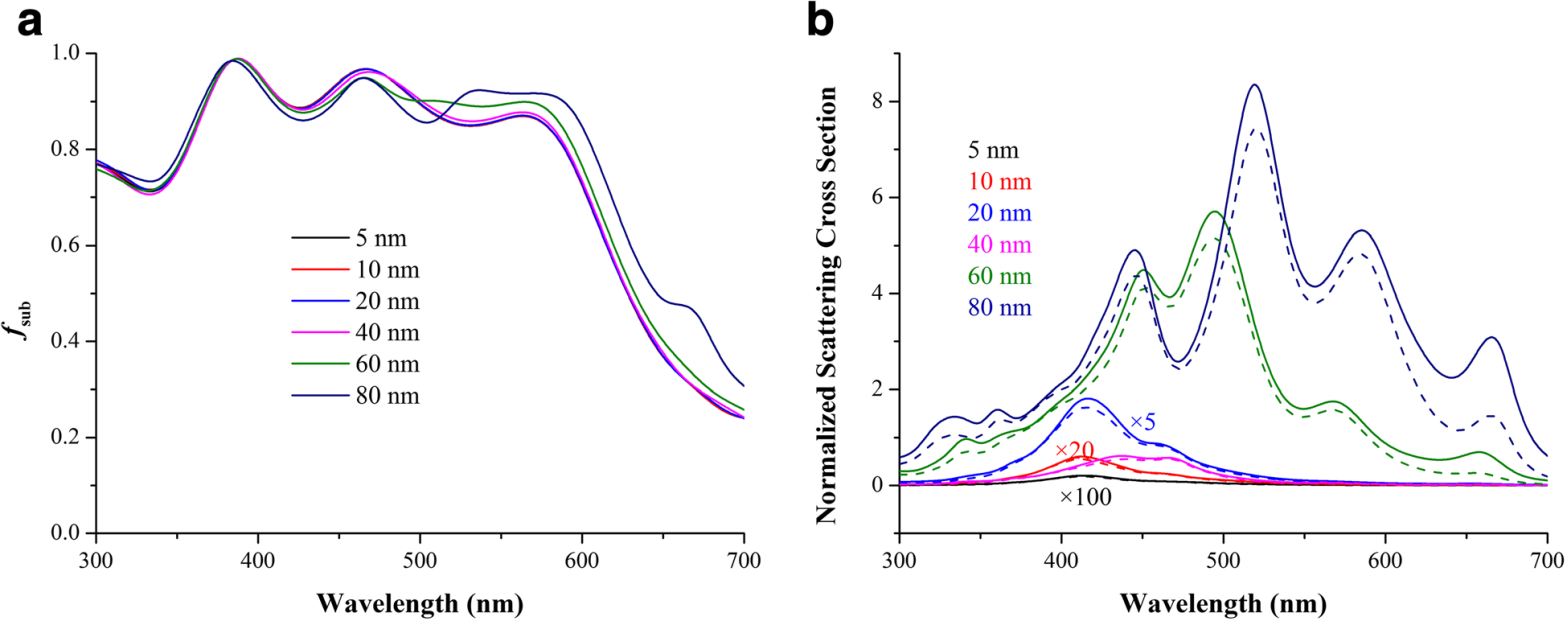
**a** The fraction of light scattered into the active layer with Al cathode presenting. **b** Total scattering cross section (solid) and cross section for light scattered into the substrate (dashed line) with Al cathode presenting, all normalized to geometrical cross sections

Al cathode in the OSCs not only serves as contact but also acts as a mirror, prolonging the pathlength of the light in an active layer. Therefore, we further investigate their scattering properties when Al cathode is presented. In the simulation, a 150-nm Al is contacted with the active layer. Figure 4 demonstrates the *f*_sub_ of various NPs. As can be seen from Fig. 4a, *f*_sub_ are significantly improved after introducing the Al mirror. However, the *Q*_scat_ of NPs are slightly influenced, as demonstrated in Fig. 4b. No matter whether the Al mirror is presented, the large-sized NPs demonstrate large scattering cross sections and a large amount of light is scattered into the substrates. Therefore, the large-sized NPs are favored when NPs are located outside of the active layer from point of optical simulation.

### NPs Located Inside of the Active Layers

**Fig. 5:**
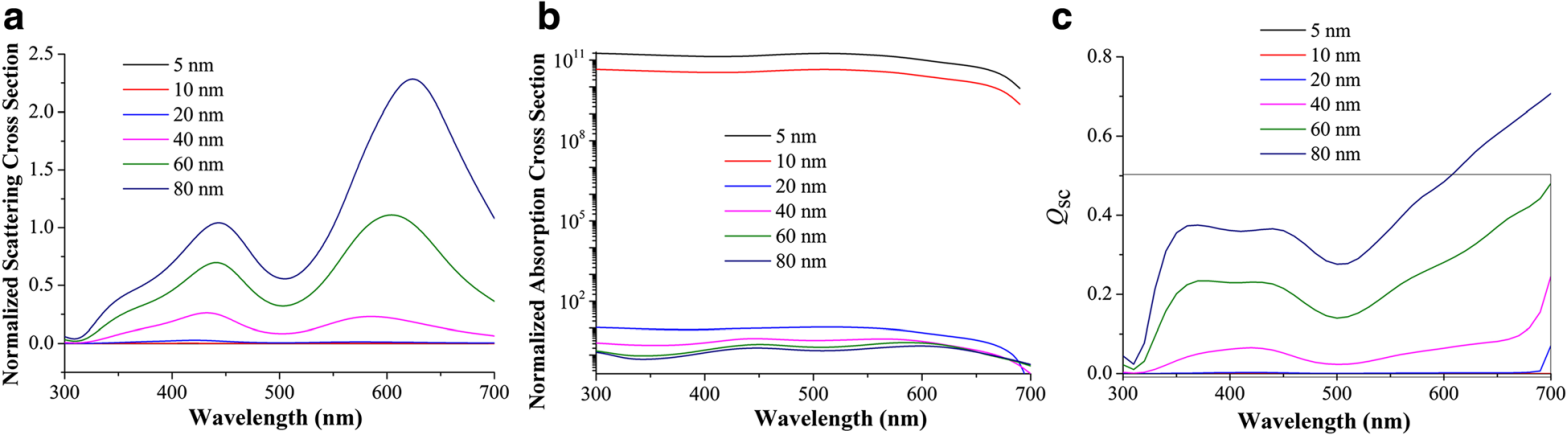
Normalized scattering cross section (**a**), absorption cross section (**b**), and scattering efficiency (**c**) of different-sized NPs in the homogeneous active layer

**Fig. 6:**
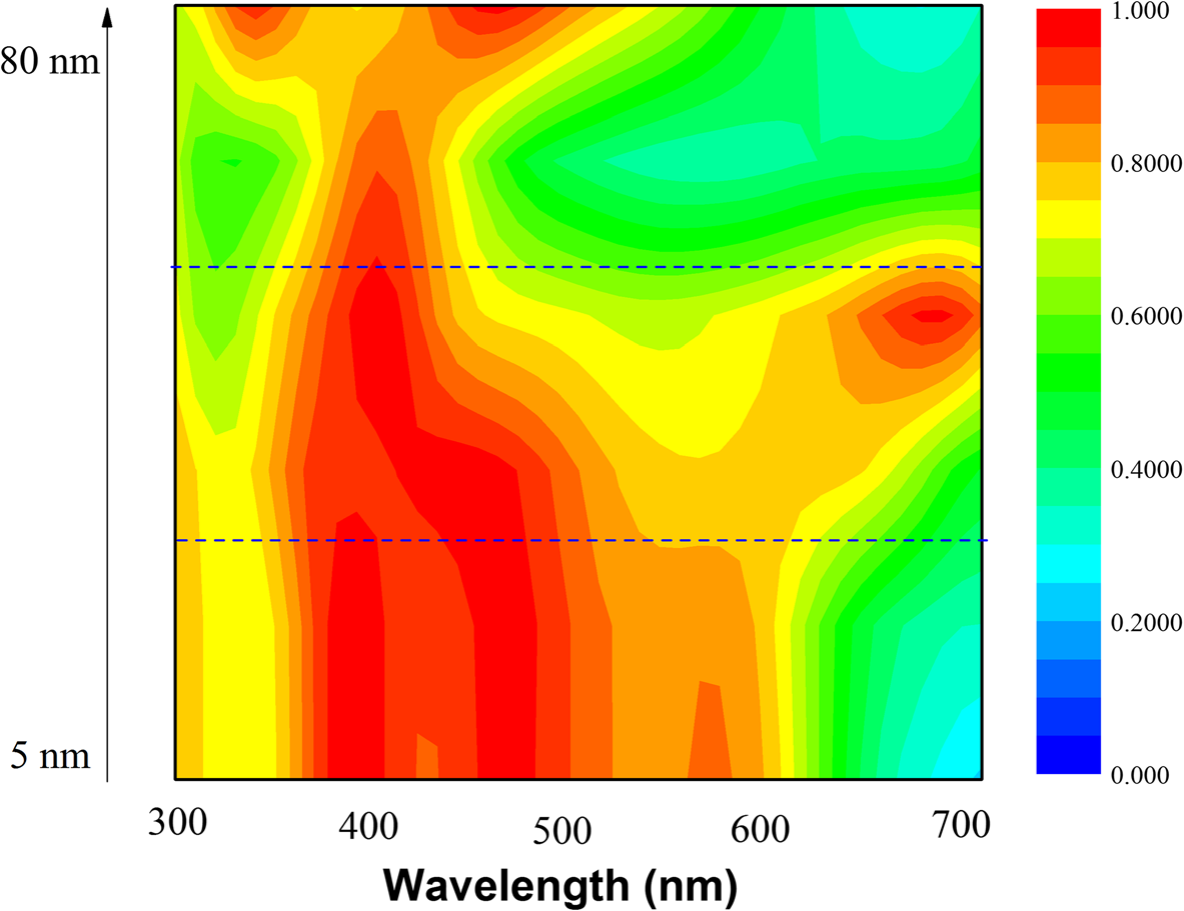
Absorption color map of different-sized NPs in the homogeneous active layer with Al cathode contacted with the active layer

While NPs are located in the matrix of the active layer, the far-field scattering and near-field enhancement influence OSCs simultaneously. For NPs with sizes well below the wavelength of light in the quasi-static limit, the scattering/absorption cross section can be interpreted by Eq. 1 [15]:

1 $$ {\sigma}_{\mathrm{sca}}=\frac{1}{6\pi }{\left(\frac{2\pi }{\lambda}\right)}^4{\left|{\alpha}_{\mathrm{sp}}\right|}^2,\cdot {\sigma}_{\mathrm{abs}}=\frac{2\pi }{\lambda}\mathit{\operatorname{Im}}\left[{\alpha}_{\mathrm{sp}}\right] $$

where *α*_sp_ is the polarizability of the sphere:

2 $$ {\alpha}_{\mathrm{s}\mathrm{p}}=4\uppi {r}^3\frac{\varepsilon_{\mathrm{m}}-{\varepsilon}_{\mathrm{s}}}{\varepsilon_{\mathrm{m}}+2{\varepsilon}_{\mathrm{s}}} $$

where the *ε*_m_ and *ε*_s_ are the permittivities of the surrounding material and of the sphere, respectively. In the case when NPs are outside, the surrounding dielectric environment is complicated and it can be calculated by approximation in a homogenous environment [15]. The *Q*_scat_ and *Q*_abs_ are plotted in Fig. 5a, b, respectively. Absorption dominates for small NPs embedding in the active layer with diameters in the range 5 to 10 nm. The plasmonic near-field is coupled to the active layer and thus increases the absorption cross section that improves exciton dissociation. However, *Q*_scat_ is much lower than that of NPs located outside of the active layer. The scattering efficiency, *Q*_sc_, defined by *Q*_scat_/(*Q*_scat_ + *Q*_abs_), is shown in Fig. 5c to evaluate either scattering or absorption dominates. *Q*_scat_ values of all NPs are no more than 0.5, suggesting absorption dominates from 300 to 700 nm. Therefore, enhanced absorption cross sections of NPs are critical when they are embedding in a homogeneous matrix, rather than scattering cross sections as in the outside case. Figure 6 demonstrates absorption spectra of active layers coupled with NPs. Small NPs have obvious absorption enhancement, but as size increases, the absorption spectra deteriorate. Although light absorption can be boosted by scattering of large-sized NPs, increased scattering cannot compensate decreased absorption, as illustrated in Fig. 5a, b. For electrical consideration, increased *Q*_abs_ in small NPs can be used in OSCs to improve exciton dissociation while large NPs give rise to recombination and exciton quenching [25]. As size increases, charge recombination and exciton quenching will become more serious [31]. Therefore, small NPs are preferred when they are covered in the active layer.

## Conclusions

In conclusion, the light-trapping abilities of Ag NPs located inside and outside of the active layer are investigated. When the NPs are located outside of the active layer, the fraction of light scattered into the active layer is crucial. Large-sized NPs have large scattering cross sections and a large amount of light is preferably scattered under the photoactive layer. On the other hand, an absorption cross section is essential when NPs are embedding in the homogeneous active layer. Small-sized NPs can boost light absorption of OSCs due to their large absorption cross sections. We believe that the results of our study might pave the way towards cost-effective OSC devices, and this approach may be applicable to OSC systems featuring other types of active materials.

## Abbreviations

FDTD: Finite-difference time-domain
ITO: Indium tin oxide
LSPR: Localized surface plasmon resonance
NPs: Nanoparticles
OSCs: Organic solar cells
P3HT: Poly(3-hexylthiophene)
PCBM: [6,6]-Phenyl-C61-butyric acid methyl ester
PCE: Power conversion efficiency
PEDOT: PSS: Poly(3,4-ethylenedioxythiophene): poly (styrene sulfonate)
PML: Perfectly matched layer
PVs: Photovoltaics
TFSF: Total-field scattered-field
